# Cortical presentation of language functions in patients after total laryngectomy: a fMRI study

**DOI:** 10.1007/s00234-020-02407-x

**Published:** 2020-04-06

**Authors:** Aleksandra Wypych, Małgorzata Wierzchowska, Paweł Burduk, Elżbieta Zawada, Katarzyna Nadolska, Zbigniew Serafin

**Affiliations:** 1 The Interdisciplinary Center for Modern Technologies, Nicolaus Copernicus University, Toruń, Poland; 2 Department of Otolaryngology, Oncology and Oral and Maxillofacial Surgery, Nicolaus Copernicus University, Collegium Medicum, Bydgoszcz, Poland; 3 Department of Radiology and Diagnostic Imaging, Nicolaus Copernicus University, Collegium Medicum, Bydgoszcz, Poland; 4 Department of Geriatrics, Nicolaus Copernicus University, Collegium Medicum, Bydgoszcz, Poland

**Keywords:** Laryngectomy, Magnetic resonance imaging, Gray matter

## Abstract

**Purpose:**

The aim of this study is to use functional magnetic resonance (fMRI) to analyse the cortical presentation of selected language functions in patients after a total laryngectomy.

**Methods:**

Eighteen patients after total laryngectomy treated with electrolarynx speech and 18 volunteers were included. The mean number of patients’ post-operative speech rehabilitation sessions was five (range of 3–8 sessions). Four paradigms were used, including noun generation, pseudoword reading, reading phrases with pseudowords, and nonliteral sign reproduction.

**Results:**

In noun, the most significant difference between the groups was the stronger activation of both lingual gyri in the volunteers. Pseudoword reading resulted in stronger activations in patients than in volunteers in the lingual gyri, the right cerebellum, the right Broca’s area, and the right parietal operculum. Reading phrases with pseudowords involved different parts of the Brodmann area 40. During nonliteral sign reproduction, there was a stronger activation of the left Broca’s area in volunteers and a stronger activation of the left premotor cortex in patients.

**Conclusion:**

This study provides evidence of altered cortical activation in response to language tasks in patients after a laryngectomy compared with healthy volunteers, which may be considered brain plasticity in response to a laryngectomy.

## Introduction

The ability to speak is one of the sophisticated features that make a distinction between humans and other animals. No wonder that how the brain controls speech has remained a subject of investigation for centuries. The pioneers of research on cerebral language functions were Pierre Paul Broca and Karl Wernicke [1, 2]. Their studies and theories had a profound effect on contemporary understanding of speech. Sakai et al. [3] commented that human language is a unique faculty of the mind. The grammaticality of a sentence, they assert, needs to be made explicit by the adoption of an appropriate theoretical framework for linguistic structures. Grammatical rules arise from the human brain so that language must be considered a subsystem of the mind, with the language system being a distinct module, which in turn possesses its own modularity or subsystems such as phonology, semantics, and syntax, which interact systematically with each other through the information flow between them [3].

However, despite the huge progress in neuroimaging, the definite location of these areas still remains controversial [4, 5]. Some authors define it as a unimodal auditory association in the superior temporal gyrus anterior to the primary auditory cortex (the anterior part of BA 22) [6]. This is the site most consistently implicated in auditory word recognition by functional brain imaging experiments [7]. Others also include adjacent parts of the heteromodal cortex in Broca’s area (BA) 39 and BA40 in the parietal lobe [8]. Recently, Ardila et al. suggested that grammar correlates with the ability to internally represent actions (verbs) depending on the functioning of BAs 44 and 45 and the brain circuits related to them [9]. Grammar is also thought to be associated with the ability to quickly carry out the sequencing of the articulatory movements required for speaking (speech praxis) [10]. Meta-analytic studies which aimed to analyse the specific contribution of different BAs to the language system identified some areas potentially related to the adoption and comprehension of language (the lexical and semantic system) and areas related to language production (the grammatical system) [9, 11].

Total laryngectomy is still the method of choice for the treatment of advanced laryngeal cancer when radiotherapy fails to preserve the organ [12]. However, after a laryngectomy, patients not only lose the ability to communicate but also fall into a kind of social exclusion [12, 13]. Voice rehabilitation is a very important part of both pre-operative and post-operative treatment. The most technically advanced speech generation technique, ELS, uses an electronic device that generates sound regulated by vibrations of the patient’s neck or cheek muscles [11]. It seems to be the most comparable to a healthy person’s speech considering acoustic parameters including fundamental frequency, maximum phonation time, and intensity of the voice [11, 14–17].

Considering the complicated and not fully understood cortical control of speech, it is obvious that a sudden inability to speak in an adult, caused by a total laryngectomy, is a vast disablement to the patient. Moreover, speech rehabilitation methods, as described above, seem to stimulate cortical function remodelling. Remodelling of both connectivity and grey matter areas is a well-known phenomenon after brain injury and the loss of peripheral functions [11, 14]. Several different language paradigms were proposed to localize speech-related brain areas [18, 19]. Word generating is one of the most commonly used paradigms that results in activation mostly at the left inferior frontal gyrus and the bilateral motor cortex [20]. One may suppose that after laryngectomy, only the activation of motor cortex may be affected. Similarly, more or less sophisticated conceptual paradigms, including pseudoword reading and repeating, and nonliteral sign reproduction [21] cortical processing should not be influenced by laryngectomy as well.

We hypothesise that a similar process takes place after a laryngectomy. To our knowledge, there are no published studies that analyse brain cortical function related to a laryngectomy. The aim of this study was to use fMRI to analyse the cortical presentation of selected language functions in patients after a total laryngectomy.

## Methods

### Participants

The study group consisted of 36 right-handed subjects, including 18 patients after a total laryngectomy (15 men and 3 women at mean age 61 ± 8 years) and 18 healthy volunteers (15 men and 3 women at mean age 57 ± 7 years). All the patients were treated with ELS and were clinically considered subjects who successfully completed post-operative speech rehabilitation. The mean number of speech rehabilitation sessions was five (range of 3–8 sessions). Exclusion criteria were as follows: psychiatric or neurological disorders, previous brain or ear surgery, pregnancy, claustrophobia, metal foreign objects or metal implants (heart stimulator, cardioverter, insulin pump, cochlear implant, CNS stimulation, prosthesis). All participants were adults and gave informed consent to participate. We obtained permission to conduct the study from the local bioethical committee.

### Scanning and language tasks

The study was conducted using a 3T MRI scanner (GE Discovery MR 750, General Electric Healthcare, Waukesha, IL, USA) and an 8-channel neurovascular head coil (HD 8-CH Neurovascular Array, General Electric Healthcare, Waukesha, Il, USA). For each participant, scanning started with a high-resolution 3D BRAVO sequence (3D T1W1, TR 8.2 ms, TE 3.2 ms, FA 12°, thickness 1.0 mm, no interslice gap, 176 slices, voxel size 1 × 1 × 1 mm). Then, the functional experiment was performed using the BOLD technique in the axial plane using an EPI sequence (TR 2000 ms, TE 28 ms, FA 90°, thickness 3.0 mm, no interslice gap, 44 slices, voxel size 3 × 3 × 3 mm). In the fMRI experiment, four paradigms were used: noun generation, pseudoword reading, reading phrases with pseudowords, and nonliteral sign reproduction. The tasks were presented in the subjects’ mother tongue (Polish) using NNL VisualSystem goggles (NordicNeuroLab AS, Bergen, Norway). Paradigms were created in Presentation (http://www.neurobs.com), a stimulus delivery and experiment control program for neuroscience.

Each language activation comprised one fMRI run per paradigm in block design experiments. Stimuli were presented as six 30 s active blocks separated by six 30 s rest pauses. Each session lasted 6 min, 10 s, including 5 dummy scans. Tasks were always presented in the same order and were of the same duration. Participants were instructed before the MRI scanning about the structure of the study and they were given examples of tasks that were different from the paradigms presented in the scanner. For each task, the volunteers were asked only to think about a word:

- In task 1, they were asked to generate nouns beginning with a given letter (e.g. T, P, R, S, K).
- In task 2, pseudonouns were presented in strings. The list of words was arranged gradually from the easiest to the most difficult in terms of structure (C means a consonant and V means a vowel): (i) two-syllable words with the CVC-CVC recording scheme, e.g. “chesnut”; (ii) polysyllable words consisting of a different number of syllables with a CV and CVC recording scheme, e.g. “chesstboard”; (iii) two-syllable words with a CCV-CV recording scheme, e.g. “prima”. Each string was displayed for 30 s and consisted of five words. The subjects were instructed to read and repeat the pseudonouns in their minds until the next string of words appeared.
- In task 3, participants were asked to read phrases consisting of single words with converted or shifted letters, e.g. “raed veihcle”, “lietr of waeter”.
- In task 4, nonliteral sign reproduction (e.g. !, +,?,:) were presented. Participants were asked to repeat the name of graphic sign in their mind.

### Data processing and analysis

Data pre-processing and analysis were performed using an FSL v. 5.0 (The FMRIB Software Library) toolkit. Images were controlled for field distortion, spike artefacts, and temporal signal-to-noise ratio. Then, EPI scans were subject to movement correction, co-registration, normalisation, segmentation, and spatial smoothing. Movement correction was conducted using a MCFLIRT package [10]. For structural and functional image registration, a FLIRT library (FMRIB’s Linear Image Registration Tool) and the MNI152 standard-space T1-weighted average structural template image were used [9]. Spatial smoothing was performed with a Gaussian filter (FWHM 4.0 mm). We also used a high-pass filter in a time domain (Gaussian-weighted least-squares straight line fitting, sigma = 50.0 S). Time series analysis was conducted using FILM software (FMRIB’s Improved Linear Model) with local correlation correction [22, 23]. The fMRI data analysis was conducted using a FEAT library v. 6.00 (FMRI Expert Analysis Tool).

Z statistic images (Gaussianized T/F) were set in terms of clusters for Z > 2.3. The cluster significance (with correction) was set at *p* = 0.05. The first five EPI scans were discarded in each data package to achieve signal balance. Cluster correction was performed on both the first-level and the second-level analysis. The first-level statistical analysis (individual data) was conducted using a general linear model (GLM). The second-level statistical analysis (group analysis) for difference assessment between healthy controls and laryngectomy patients was conducted using a FLAME library (FMRIB’s Local Analysis of Mixed Effects), which is a type of variance test for modelling and estimating the random-effects component of the measured inter-session mixed-effects variance in a full Bayesian network. FLAME 1 option with mixed-effects model was applied. Statistical analysis was made by the unpaired sample *t* test. Each task was analysed in two variants: activation stronger in controls than in patients and activation stronger in patients than in controls. Significant clusters were labelled based on Juelich Histological Atlas [24].

## Results

None of participants terminated the MRI examination and none of exams were rejected due to artefacts.

Results showed a stronger activation of the left visual cortex V3V and come part of the right visual cortex V2 in controls compared with patients during a noun generation task. Conversely, laryngectomy patients presented stronger activation of the right visual cortex V1, another part of the right visual cortex V2, the right inferior parietal lobule, the left cingulum, and the right premotor cortex (Table 1). On the other hand, in response to task 2, a stronger activation in volunteers was seen for visual cortex V3 and a stronger activation in patients for the right visual cortex V2, the left visual cortex V4, and the right Broca’s area (Table 2).

**Table 1:** Response to task 1

Juelich histological atlas	*Z* value	*P* value	x	y	z
Activation stronger in healthy volunteers than in laryngectomy patients					
Left visual cortex V3V	2.86	< 0.0000	53	22	33
Right visual cortex V2	2.79	0.0002	41	22	33
Right visual cortex V3	2.53	0.0031	29	21	32
Right cingulum	2.46	0.0042	42	40	49
Left secondary somatosensory cortex / parietal operculum OP4	2.43	0.0369	67	58	42
Visual cortex V1	2.43	0.0369	40	21	38
Right Broca’s area	2.40	0.0435	24	72	39
Right secondary somatosensory cortex/parietal operculum OP1	2.39	0.0435	18	50	47
Activation stronger activated in laryngectomy patients than in healthy volunteers					
Right visual cortex V1	2.63	0.0002	37	17	38
Right visual cortex V2	2.61	0.0027	35	21	32
Right inferior parietal lobule Pga	2.47	0.0028	18	35	52
Left cingulum	2.42	0.0032	48	55	52
Right premotor cortex	2.37	0.0036	37	55	67
Left premotor cortex	2.32	0.0413	51	56	69

**Table 2:** Response to task 2

Juelich histological atlas	*Z* value	*P* value	x	y	z
Activation stronger in healthy volunteers than in laryngectomy patients					
Visual cortex V3	2.79	0.0042	34	23	32
Activation stronger activated in laryngectomy patients than in healthy volunteers					
Right visual cortex V2	2.75	< 0.0000	38	23	33
Left visual cortex V4	2.55	< 0.0000	56	24	33
Right Broca’s area	2.45	0.0009	18	74	39
Right secondary somatosensory cortex/parietal operculum OP1	2.35	0.0019	17	51	47
Left visual cortex V1	2.33	0.0282	51	18	37
Left visual cortex V2	2.32	0.0313	49	16	33

**Fig. 1:**
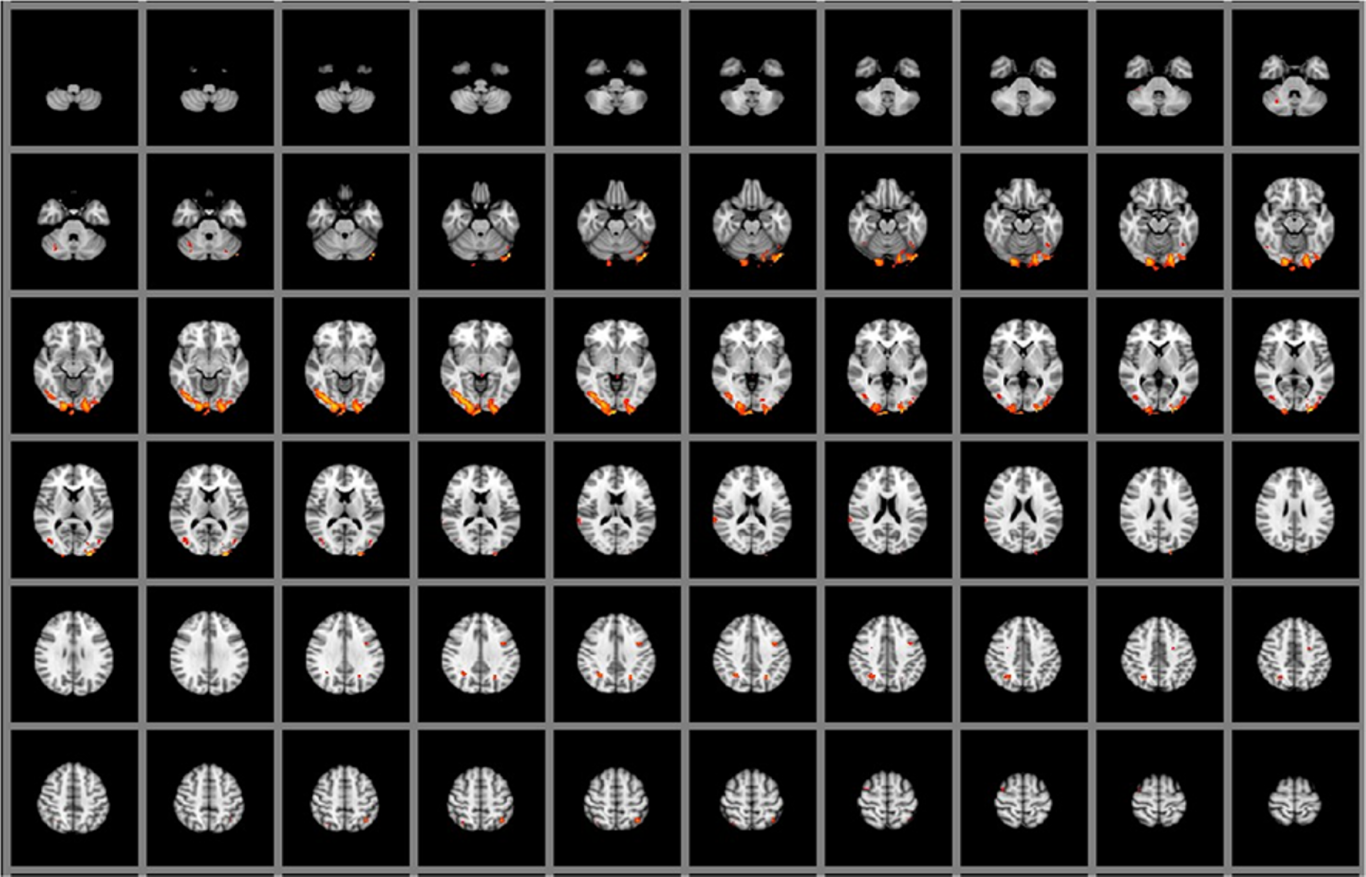
Graphical presentation of the brain areas that were significantly stronger activated in laryngectomy patients than in healthy volunteers in response to task 3

Task 3 resulted in the strongest cortical activation in patients. The left Broca’s area, the left anterior intra-parietal sulcus, and visual cortex (V3, V4) were strongly activated in patients, whereas V1 and V2 visual cortex, the left primary somatosensory cortex, and the left premotor cortex presented stronger response in controls (Table 3, Fig. 1). Finally, during nonliteral sign reproduction, controls activated more the left Broca area than patients, while patients activated more the left premotor cortex than controls (Table 4).

**Table 3:** Response to task 3

Juelich histological atlas	*Z* value	*P* value	x	y	z
Activation stronger in healthy volunteers than in laryngectomy patients					
Left Broca’s area	3.10	< 0.0000	71	75	42
Left anterior intra-parietal sulcus hip2	2.57	< 0.0000	67	44	54
Right visual cortex V3	2.53	< 0.0000	34	23	32
Right visual cortex V4	2.43	0.0006	29	25	31
Left inferior parietal lobule pft	2.39	0.0032	72	50	54
Right visual cortex V2	2.32	0.0034	37	17	38
Activation stronger activated in laryngectomy patients than in healthy volunteers					
Left visual cortex V1	2.73	0.0010	49	15	37
Right visual cortex V2	2.65	0.0002	37	21	32
Left primary somatosensory cortex	2.58	0.0035	71	55	58
Left visual cortex V2	2.46	0.0028	49	16	33
Left premotor cortex	2.46	0.0020	51	56	69
Right premotor cortex	2.38	0.0252	38	55	71
Left primary somatosensory cortex	2.38	0.0392	70	52	60

**Table 4:** Response to task 4

Juelich histological atlas	*Z* value	*P* value	x	y	z
Activation stronger in healthy volunteers than in laryngectomy patients					
Left visual cortex V2	2.65	< 0.0000	52	15	33
Left visual cortex V1	2.53	< 0.0000	48	15	37
Right visual cortex V3	2.53	< 0.0000	30	22	32
Left Broca’s area	3.42	0.0035	68	67	47
Right inferior parietal lobule Pga	3.41	0.0002	23	35	50
Right visual cortex V1	2.40	< 0.0000	35	26	38
Left inferior parietal lobule pgp	2.39	0.0132	62	27	50
Activation stronger activated in laryngectomy patients than in healthy volunteers					
Left visual cortex V2	2.56	0.0057	55	13	33
Left premotor cortex	2.48	0.0028	57	56	69
Right secondary somatosensory cortex/parietal operculum OP1	2.42	0.0433	23	49	47
Left visual cortex V1	2.41	0.0008	46	18	37
Right visual cortex V2	2.39	0.0068	37	17	32

## Discussion

To our knowledge, this is the first study published presenting differences between patients after a laryngectomy and healthy volunteers in cortical activation in response to language tasks.

Tasks resulted in activations at different levels of the visual cortex but no clear pattern could be defined in both volunteers and patients. The only difference was activation of the V3 visual cortex in volunteers in most of the experiments. Despite intensive investigation, the precise location and function of the third visual cortex remains a matter of debate [25, 26]. Generally, it is considered to play a role in the processing of motion, either global or coherent [25]. Based on the construction of tasks used in this study, the observed stronger activation of the V3 cortex in healthy subjects remains unclear.

Noun generation (task 1) requires semantic categorisation and, thus, makes great demands on semantic processing [27]. The most significant difference between the groups was the stronger activation of both lingual gyri in the volunteers. These areas are responsible for semantic categorisation, word retrieval, word generation, and single letter processing [28–31] so their activation presents a proper function. On the other hand, patients after a laryngectomy presented a stronger activation of the right angular gyrus, the left anterior cingulate gyrus, and the bilateral premotor cortex. The angular gyrus is surrounded by secondary somatosensory, visual and auditory cortical areas and is essential in the multimodal, highly complex synthesis of information [32]. Thus, it can be considered an adjuvant cortical area activated in patients. The anterior cingulum was linked to many different functions, including semantic and phonological verbal fluency [33]. However, in patients after a laryngectomy, activation of this area may be also linked to its role in cognitive and motor inhibition, motor imagery as well as in motor preparation and planning [34, 35]. The premotor cortex, which is also connected with multiple functions, was activated for word generation in other studies [27]. Basic functions of this area include motor sequencing, movement planning, and imagination of movement [36, 37]. These functions may require stronger activation when oral speech has to be replaced by artificial phonation. The left premotor cortex is also responsible for speech initiation and speech motor programming [15, 38], which again require more effort after a laryngectomy.

Pseudoword reading (task 2) resulted in stronger activations in patients than in volunteers in the lingual gyri, the right cerebellum, the right Broca’s area, and the right parietal operculum (OP1). Previous studies indicated that pseudoword reading involved the left fusiform gyrus, the left angular gyrus, and the left middle temporal gyrus for lexical and semantic processing. Furthermore, spelling-sound conversion was located in the left inferior parietal gyrus, and phonological output in the left inferior frontal gyrus [39]. Hauck et al. also found significant activation in the left inferior parietal gyrus, which confirms our results. In general, our results show more complicated processing of pseudoword reading after a laryngectomy. Activation of OP1 has been linked to literal sentence comprehension, and word imageability [10, 40]. The contribution of the cerebellum to pseudoword processing is less clear. Guediche et al. postulated a functional network between the cerebellum and language-related regions in the temporal and parietal lobes contributing to sensorimotor adaptation. They stated that cerebro-cerebellar interactions may support supervised learning mechanisms that rely on sensory prediction error signals in speech perception [41]. Therefore, cerebellar activation in our patients may be a feature of brain plasticity in response to a laryngectomy.

Reading phrases with pseudowords (task 3) was a more complicated paradigm and involved different parts of the Brodmann area 40 L that is responsible for more elaborate semantic processing [42]. Apart from the activations discussed above, an interesting observation was a much stronger left Broca’s area activation in volunteers than in patients, which again underlines altered speech processing after a laryngectomy. On the other hand, a stronger response from the left primary somatosencory cortex and the bilateral premotor cortex was observed in patients.

Cortical representation of nonliteral sign reproduction (task 4) remains a matter of debate. A meta-analysis by Rapp et al. indicated that a predominantly left lateralised network, including the left and right inferior frontal gyrus, the left, middle, and superior temporal gyrus, and medial prefrontal, superior frontal, cerebellar, parahippocampal, precentral, and inferior parietal regions, was important for nonliteral expressions [43]. On the other hand, Yang et al. concluded that there is flexible involvement of the sensory-motor system in abstract concept processing, which depends on semantic features of the language stimuli and links between abstract and literal meanings [12]. In our study, the most significant differences between the groups again included a stronger activation of the left Broca’s area in volunteers and a stronger activation of the left premotor cortex in patients.

Some limitations of the current study have to be addressed. Firstly, the time of rehabilitation after a laryngectomy varied in our study group between three and eight learning sessions. We believe that the extent and the efficiency of speech processing plasticity in the brain may be dependent on the duration of rehabilitation and therefore might influence the results. This hypothesis still needs confirmation. On the other hand, the number of sessions to finish rehabilitation depended on individual abilities of patients. At inclusion to the study, all the patients had finished their speech rehabilitation with a positive outcome. Therefore, they were clinically diagnosed as positively rehabilitated. Considering this diagnosis, the group may be considered homogenous. Secondly, study participants were not selected according to education and profession, which also may have an impact on language processing. Thirdly, a more detailed analysis of inter-subject variability would be necessary to find other possible covariates of the outcome. For instance, differences in activations may be related to cognitive abilities of subjects, which are crucial to understand if changes in cortical activations are compensatory or maladaptive. As results of the current preliminary study appeared encouraging to us, we are now starting larger program, including cognitive and psychological testing. Therefore, further investigation is necessary involving more sizeable and carefully composed study groups to validate the current findings.

In conclusion, this study provides the first evidence of altered cortical activation in response to language tasks in patients after a laryngectomy compared with healthy volunteers, which may be considered brain plasticity in response to a laryngectomy.
